# Bone Control of Muscle Function

**DOI:** 10.3390/ijms21041178

**Published:** 2020-02-11

**Authors:** Giulia Battafarano, Michela Rossi, Francesco Marampon, Salvatore Minisola, Andrea Del Fattore

**Affiliations:** 1 Bone Physiopathology Unit, Genetics and Rare Diseases Research Area, Bambino Gesù Children’s Hospital, 00165 Rome, Italy; giulia.battafarano@opbg.net (G.B.); michela1.rossi@opbg.net (M.R.); 2 Department of Radiotherapy, Policlinico Umberto I, “Sapienza” University of Rome, 00151 Rome, Italy; francesco.marampon@uniroma1.it; 3 Department of Clinical, Internal, Anesthetic and Cardiovascular Sciences, “Sapienza” University of Rome, 00151 Rome, Italy; salvatore.minisola@uniorma1.it

**Keywords:** bone, muscle, osteocalcin

## Abstract

Bone and muscle represent a single functional system and are tightly connected to each other. Indeed, diseases characterized by alterations of muscle physiology have effects on bone remodeling and structure and vice versa. Muscle influence on bone has been deeply studied, and recent studies identified irisin as new molecule involved in this crosstalk. Muscle regulation by bone needs to be extensively investigated since in the last few years osteocalcin was recognized as a key molecule in the bone–muscle interaction. Osteocalcin can exist in two forms with different degrees of carboxylation. The undercarboxylated form of osteocalcin is a hormone released by the bone matrix during the osteoclast bone resorption and can bind its G-protein coupled receptor GPRC6A expressed in the muscle, thus regulating its function. Recently, this hormone was described as an antiaging molecule for its ability to regulate bone, muscle and cognitive functions. Indeed, the features of this bone-related hormone were used to test a new therapeutic approach for sarcopenia, since injection of osteocalcin in older mice induces the acquirement of physical abilities of younger animals. Even if this approach should be tested in humans, osteocalcin represents the most surprising molecule in endocrine regulation by the skeleton.

## 1. Introduction: Muscle and Bone as an Integrated System

The musculoskeletal system protects our internal organs and gives us support and the ability to move. Muscle and bone define a single functional system and are connected each other during the life. They derive from somites originating from the paraxial mesoderm, synergically develop, operate together and simultaneously age. Indeed, during intrauterine development they have the same progenitors [1]. The beginning of skeletogenesis is represented by the migration of mesenchymal cells derived from embryonic lineages to the sites of bone development. Here, mesenchymal cells condensate and act as multipotent cells giving rise to progenitors and tissue-specific stem cells with more dedicated functions; then, they can differentiate into chondrocytes for endochondral bone formation, or into osteoblasts for direct ossification [2,3]. The definition of skeletal stem and progenitor cells evolved during last decades. Indeed, the identification of stem cell populations in the bone marrow was made in the 1960s. In 1980 the CFU-F (colony-forming units-fibroblast) cells were identified as responsible for heterotopic ossicle formation. Later, mesenchymal stem cells (MSCs), or more accurately skeletal stem cells (SSCs), were identified as the subset of CFU-F cells with a self-renewal ability and multipotency, as demonstrated by their capability to differentiate into osteoblasts, chondrocytes and adipocytes in vitro [3]. Since skeletal stem cells are not only involved in bone development but also in its maintenance and repair, they are located in several sites such as the perichondrium of fetal bone, periosteum and bone marrow space in adults [3]. The muscle also derives from somites, and their cells undergo delamination, migration, proliferation and determination, leading to the precursor cells that finally will give rise to muscle formation via differentiation [1]. As for bone, also in adult muscle a niche of stem cells is preserved and it is represented by the satellite cells. These cells are dormant progenitors located at the periphery of skeletal myofibers, and they are inducible to proliferate and to differentiate following injury. It has been also suggested that other cells can enter into the satellite cell positions from other sources, like bone marrow, to participate in muscle regeneration [4].

It was demonstrated that adult animal myoblasts were able to transdifferentiate into osteogenic cells and that satellite cells can become osteoblasts and osteocytes during the healing process [5,6,7]. In vitro experiments performed using muscle C2C12 cells confirmed the ability of myoblasts to transdifferentiate into bone cells [8]. However, the ability of osteoblasts to differentiate into muscle cells has not been demonstrated. 

The musculoskeletal system has a schizophrenic feature since it has two identities: it is a victim of aging and it is a gatekeeper delaying aging.

During growth and development, bone and muscle acquire mass and strength [9]. The muscle–bone unit is influenced by sex, weight, aging and physical activity. Particularly, Summik et al. showed that both bone mass content (BMC) and muscle cross-sectional area (Ma) were greater in men than in women. Moreover, the BMC/Ma ratio is constant during life, and it is higher in women than in men [10]. There is a positive correlation between body mass and bone mass. An increase of body mass index is usually correlated with an increase of bone mineral density (BMD), resulting in reduced fracture risk. However, the body composition is also important. The different composition in lean and fat mass can differently improve BMD [11]. At the age of 50–60 years, sarcopenia often occurs with reduction of muscle strength, power and resistance [12,13]. Sarcopenia is clinically evident when the ratio between appendicular skeletal muscle mass and height is >2 standard deviations below the level observed in age- and sex-matched controls [14]. The principal evidence of sarcopenia is the reduction of fiber number, but also atrophy (particularly in fiber type II) is involved [13]. At the same time the cortical bone can lose its elastic modulus, resistance and stiffness, leading to osteoporosis [15,16]. It is not clear if one condition predates the other one. According to the mechanical thesis supporting that muscular contraction stimulates bone formation and regeneration [17,18,19], muscle loss should begin before bone loss. Patients affected by dystrophy also showed reduced bone mass and increased bone frailty and fractures [20,21]. At the same time, in vivo experiments demonstrated that muscle paralysis is associated with bone loss phenotype [22], and alteration of BMD was reported in humans after spinal cord injury [23]. However, it was observed that some patients displayed reduced bone mass independently of sarcopenia development, or they received a diagnosis of sarcopenia after bone loss occurred [24,25].

Muscle and bone interact anatomically and biochemically. They are coordinated by common pathways and coordinate each other by paracrine signals known as myokines and osteokines. This tight interaction between the two systems is relevant in an evolutionary context. Indeed, in case of danger, bone protects internal organs from trauma, and it cooperates with muscle to trigger the escape of the animal. Indeed, Berger et al. recently demonstrated that the acute stress response is mediated by the skeleton, particularly by the stress hormone osteocalcin [26].

## 2. Mechanical Loading and Physical Activity

Muscle represents the primary source of mechanical stimuli for bone. Indeed, through muscles, the peak loads reach the bone, thus generating the highest strains. Moreover, muscles provide low-magnitude stimuli in the high-frequency domain [27]. The mechanism by which bone tissue enhances its strength and mass in response to the mechanical loads carried by the muscle is known as functional adaptation. The first description of bone as a mechanical system was in Wolff’s law, which has been redefined by Frost [28] as the capability of bone to respond to a mechanical load modulating its mass and shape. Increase or decrease of mechanical stimuli induces a shift of its metabolic equilibrium in support of anabolic (osteogenesis) or catabolic (resorption) processes. 

Bone’s adaptive reaction to mechanical stimuli implies its capability to translate mechanical information, like strain, to biological instructions. The mechano-transduction of bone is actually far from being fully understood.

Physical activity has beneficial effects on the musculoskeletal system and on bone health. Although aerobic exercise is important in maintaining overall health, resistance training has more positive effects in maintaining or improving bone mass and architecture, and it is safe and feasible for older people [29]. Moreover, appropriate training could be very important to reduce the risk of falls and fall-related consequences that impact the functional ability and the quality of life in patients with bone loss diseases.

On the contrary, immobilization, bed-rest or microgravity negatively affect bone and muscle, and they induce sarcopenia and osteopenia, which are characterized by a simultaneous decrease of anabolic signaling in favor of catabolic process [30,31,32,33]. Prolonged periods of disuse induce bone loss more rapidly than that observed with normal aging or even with menopausal osteoporosis [34,35]. Disuse osteopenia shows alterations in bone cross-sectional geometry and cancellous microarchitecture. Moreover, the increase in marrow fat is observed in osteoporosis due to a shift of stromal cell population toward adipogenesis at the expense of osteoblastogenesis [36,37,38,39].

In addition to the mechanical influence, many other factors simultaneously affect bone and muscle such as genetics and endocrine signals. There is evidence that muscle and bone share genetic determinants and the pleiotropic effect has to be considered. Indeed genetic polymorphisms of genes encoding androgen and estrogen receptors, IGF-I (insulin-like growth factor), vitamin D receptor and LRP-5 (low-density lipoprotein receptor-related protein 5) may be candidates affecting osteoporosis and sarcopenia [40]. Different hormones can influence the musculoskeletal system, like sex hormones, which define sex dimorphism [41]; the GH (growth hormone)/IGF-1 axis, which is the major actor for muscle maintenance and hypertrophy, and it is also central to maintain bone homeostasis throughout life [42,43,44]; and vitamin D3, which acts directly and indirectly via calcium/phosphate metabolism on muscle and bone [45]. Although GH directly influences skeletal cells, its effects are mainly mediated by IGF-1, which enhances the function of differentiated osteoblasts and bone formation [46]. Particularly, Di Girolamo and coworkers demonstrated, by selective disruption of IGF-1R in osteoblasts, that osteoblast apoptosis is prevented by GH independently of IGF-1R in vitro, whereas the in vivo effects are likely to be mediated by IGF-1 coming from circulation and/or paracrine production [47]. In skeletal muscle, reduced efficiency of the GH/IGF-1 signaling pathway is observed during aging, as confirmed by reduction of GHR (GH receptor), IGF-1 mRNA expression and CSA of type II muscle fibers observed in biopsies of older men [48]. In age-related sarcopenia, myostatin also may play a role; a significant, negative relation between GHR and myostatin levels has been identified in healthy older adults [49]. 

Since it is now well established that bone is an endocrine organ, many studies have been published to underline that bone is able to regulate muscle function. It was demonstrated that patients affected by osteogenesis imperfecta, characterized by mutations of *COL1A1* and *COL1A2,* displayed bones more prone to fracture and muscle weakness without evidence of muscle myopathy. These results were observed both in humans and in mice and suggested that suffering bone releases factors that affect muscle functions or weaker bones are characterized by defective secretory abilities [50].

## 3. Bone and Muscle Cross-Talk: Osteokines and Myokines

**Figure 1:**
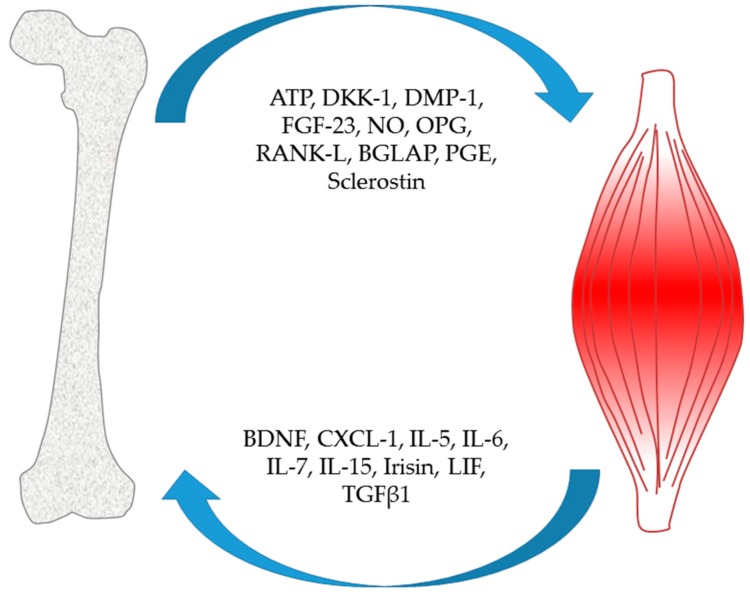
Muscle and bone interaction. Muscle and bone represent two endocrine organs that communicate with each other by the secretion of soluble factors. Factors released from the bone that influence muscle function: ATP (adenosine triphosphate); DKK-1 (Dickkopf-1); DMP-1 (dentin matrix acidic phosphoprotein 1); FGF-23; NO (nitric oxide); OPG (osteoprotegerin); RANKL; BGLAP (gamma-carboxyglutamic acid-containing protein) osteocalcin; PGE (prostaglandins); sclerostin. Muscle is able to stimulate bone by brain-derived neurotrophic factor (BDNF), CXCL1 (chemokine (C-X-C motif) ligand 1), interleukins, irisin, LIF (leukemia inhibitory factor) and TGFβ1.

The close functional relationship between muscle and bone needs intimate cross-talk that is not limited to bone mass regulation via mechanotransduction, but it involves also paracrine and endocrine signals. Factors involved in this interaction are listed in Figure 1.

Skeletal muscle, as an endocrine organ, as well as bone produce several secreted factors referred to as myokines. This list of molecules includes myostatin, IL-6 (interleukin 6), IL-8, IL-15, LIF (leukemia inhibitory factor), BDNF (brain-derived neurotrophic factor), follistatin-like 1, FGF21 (fibroblast growth factor 21) and irisin acting in autocrine, paracrine or endocrine manners [51]. Many of these myokines can significantly influence bone repair and bone metabolism. 

Myostatin belongs to the TGF beta superfamily and regulates muscle mass. Mice overexpressing myostatin show a decrease of bone mass, while myostatin-deficient animals display muscle hypertrophy [52]. Myostatin affects bone tissue; indeed, mesenchymal stem cells of myostatin null mice showed an increase in osteoblast differentiation [53]. In contrast, myostatin enhances the expression of RANKL (receptor activator of nuclear factor kappa-Β ligand), stimulating osteoclast differentiation and activity. These findings suggest that myostatin exerts negative effects on bone mass through decreased bone formation and enhanced resorption. Myostatin might be a crucial target for sarcopenia and osteoporosis. A soluble myostatin decoy receptor, ACVR2B-Fc, was generated, and its administration enhanced hind limb skeletal muscle weight in osteogenesis imperfecta and stimulated muscle mass and bone formation in postmenopausal women [54,55].

IL-6 is abundantly expressed in muscle, and it is released in response to exercise and muscle contraction. The muscle-induced and transient expression of IL-6 can act in an autocrine or paracrine manner, stimulating anabolic pathways associated with muscle growth, myogenesis and with regulation of energy metabolism [56]. The effects of IL-6 on bone cells lead to the alteration of bone remodeling activity. It was demonstrated that 10-day-old overexpressing IL-6 mice (NSE/hIL-6 mice) showed an osteopenic phenotype due to reduced osteoblast differentiation and mineralization and increased osteoclastogenesis [57]. 

In vitro and in vivo studies described myokine IL-15 and its receptor, IL-15Rα, as anabolic/anti-atrophy agents [58,59]. Moreover, expression of IL-15 mRNA is up-regulated along myoblast differentiation [60]. In humans, circulating IL-15 is elevated in response to a single session of resistance exercise in untrained and trained states [61]. Elevated IL-15 receptor alpha (IL15Rα) levels are found in the synovial fluid of patients affected by rheumatoid arthritis and other chronic inflammatory diseases that are associated with bone loss [62,63]. Indeed, IL-15 has a direct effect on bone cells. Djaafar et al. demonstrated that the absence of IL-15 signaling impairs osteoclast activity and protects against trabecular bone loss in ovariectomized mice [64]. Regarding the osteoblast side, it was shown that IL-15Rα decreased bone mineralization in vivo and in isolated primary osteogenic cells. IL-15Rα-/- osteoblasts also express reduced Rankl/Opg mRNA ratio, indicating defective osteoblast/osteoclast coupling [65].

Jahn K et al. demonstrated that secreted factors released from C2C12 myotubes were able to increase the viability of MLO-Y4 osteocytes treated with dexamethasone. The authors showed that these effects were dependent on the contractile motility of muscles [66].

Irisin is the last myokine that was discovered, and it is released following exercise [67,68]. Irisin increases osteoblast differentiation and mineralization, whereas it suppresses osteoclast differentiation [67]. In vivo studies confirmed its positive effects on bone mass since it increased cortical bone mass by stimulating bone formation and inhibiting osteoclast resorption [69]. Previous studies were performed using murine cells and models. The relevance of irisin in humans has been recently demonstrated. Indeed the first method to detect circulating irisin was based on enzyme-linked immunoassays using antibodies with a poor specificity for the protein [70]. However, Jedrychowski et al. used mass spectrometry to reveal irisin in human serum and confirmed increased levels after exercise [71].

The linkage from bone to muscle is less characterized than the humoral factors linking muscle to bone. However IGF-I, MGF (mechano growth factor), VEGF (vascular endothelial growth factor) and HGF (hepatocyte growth factor) may be anabolic and metabolic factors that affect muscle and are produced by bone cells [72]. For example, it was demonstrated that MGF is highly expressed in osteoblasts in response to mechanical stimuli, promoting the proliferation and the migration of osteoblasts. MGF has been shown to stimulate muscle stem cells (satellite cells) to re-enter cell cycle and proliferate, resulting in new muscle cells to replace injured fibers [73].

An endocrine factor released by bone is FGF23 (fibroblast growth factor 23). This growth factor is mainly released by osteocytes, and it regulates phosphate mineral homeostasis targeting distant organs such as the kidney. At the same time, FGF23 acts on the parathyroid gland to decrease PTH (Parathyroid horome) secretion [74]. Recent papers demonstrated that elevated FGF23 is associated with increased risk of heart disease and impaired vascular function [75,76]. However, the effects of FGF23 on skeletal muscle function is not known.

RANKL is produced by stromal cells and immune cells. In bone its function is to regulate osteoclastogenesis and osteoclast survival and activity by binding to its receptor RANK expressed on osteoclasts and their precursors [77,78]. However, RANK is expressed also by skeletal muscle more in oxidative muscle such as the soleus (an predominantly composed of type I fibers) than in the gastrocnemius (a mix of type I and II fibers) [79]; RANKL–RANK interaction regulates Ca2+ storage. This pathway could be involved in the muscle weakness of dystrophic mdx mice, since it was shown that RANK deletion, or treatment with the RANKL receptor decoy osteoprotegerin in dystrophy, enhanced muscle strength in mice [80]. In humans, the beneficial effects of RANKL on skeletal muscle function have been demonstrated with the fully human monoclonal antibody Denosumab in rare cases of facioscapulohumeral muscular dystrophy [81].

Osteocalcin is also involved in cross-talk between muscle and bone, consequentially to mechanical load, and stimulates the increase of muscle mass and function [82].

### 3.1. Osteocalcin

Osteocalcin (Ocn) is a 49 amino acid polypeptide protein. It is the most abundant noncollagenous protein within the bone matrix, representing about 15%, mainly produced by the osteoblasts (30), and it is thought to regulate mineralization [83]. It has been named osteocalcin because of its presumed involvement with calcium [83]. Osteocalcin was routinely used as a marker of bone formation. However, it has many features resembling a hormone:

(1) It is produced as a pro-peptide that is cleaved by osteoblasts before its secretion. The human osteocalcin gene *BGLAP* encodes a pre-pro-protein of 98 amino acids. To obtain the mature form of this protein, sequential cleavages to remove signal- and pro-sequences are necessary;
(2) In the circulation its concentration is ng/mL;
(3) Its levels are regulated by a circadian rhythm. In humans, osteocalcin levels are very low in the morning, and they start to rise in the afternoon and reach a peak in the night.

During the 1970s, Hauschka and Reid developed an analytical procedure for γ-carboxyglutamate (γCGlu) and focused their studies on chicken bone osteocalcin, defining it as a γ-carboxylglutamic acid-containing protein [84]. Indeed, human osteocalcin requires γ-carboxylation of three residues (at positions 17, 21 and 24) [83,85].

Osteocalcin synthesis involves vitamins K, D and A [86,87,88]. Indeed, γ-glutamyl carboxylase utilizes vitamin K as a cofactor [86]. Moreover, since VDREs (vitamin D response elements) are located near the *BGLAP* gene promoter, osteocalcin transcription is induced by 1,25-dihydroxylated vitamin D3 [89]. Finally, retinoic acid involvement in the regulation of osteocalcin gene expression has also been demonstrated [90].

The predominant osteocalcin form available in serum is the undercarboxylated (ucOC) form. ucOC can come from osteoclast matrix resorption due to the decarboxylation occurring in acid pH conditions [91]. Indeed, to resorb bone matrix osteoclasts, the resorption lacuna is acidified by the secretion of H + by vacuolar proton pump V-H + ATPase. This allows the dissolution of inorganic bone matrix and the decarboxylation of osteocalcin. However, a small amount of ucOC, released by the osteoblasts, is not fully carboxylated [26]. Indeed, it was recently demonstrated that during an acute stress response (ASR), a glutamate-dependent mechanism in osteoblasts inhibited GGCX gamma-glutamyl carboxylase activity, stimulating the release of active hormone. These effects observed in ASR are not mediated by the bone resorption ability of osteoclasts since this increase of ucOC was also observed in *oc/oc* osteopetrotic mice with loss of function mutation of *tcirg1*, encoding the a3 subunit of V-H + ATPase. The same modulation of undercarboxylated osteocalcin was observed in wild-type animals treated with the bone resorption inhibitor alendronate and exposed to stressor signals [26]. Under-carboxylated osteocalcin can enter the circulation and control several physiological processes, such as energy metabolism, via increasing insulin secretion and sensitivity [91], anti-tumor immunity [92], male fertility [93,94], cognition and brain development [95], muscle growth [96] and mediation of an acute stress response [26].

### 3.2. Osteocalcin and Physical Activity

The first metabolic role associated with undercarboxylated osteocalcin was identified by Karsenty’s research group and focused the attention on insulin sensitivity and diabetes risk [91]. Since muscle is the major site for energy expenditure [97], it plays an important role in glucose uptake and utilization [98]. Furthermore, insulin does not promote glucose catabolism in muscle, and its circulating levels decline during exercise [99,100] suggesting that catabolism occurring during exercise in muscle is likely driven by other hormones [101]. Thus, the interest on the potential metabolic role of ucOC acting on muscle has increased during recent years. It has been demonstrated that osteocalcin signaling is involved in myofiber adaptation to exercise [96,102]. Mera et al. showed that circulating osteocalcin levels were regulated by exercise [102]. In young adult (3-month-old) wild-type mice, exercise leads to increased total and undercarboxylated osteocalcin serum levels [102]. This increase is associated with reduced insulin levels and could be due to bone resorption, since high C-terminal telopeptide of type 1 collagen (CTX) serum levels are also observed after running. Enhancement of osteocalcin serum levels after exercise has been shown also in adult women [102]. Moreover, the authors described how osteocalcin circulating levels decrease during aging, when exercise capacity declines. They demonstrated that the highest levels are reached during adolescence for both women and men, whereas during adulthood total osteocalcin begins to decrease, reaching the lowest levels in women at the age of 30 years and in men at the age of 50 years. This age-dependent regulation of osteocalcin levels can explain why the increase in serum osteocalcin after exercise is higher in younger animals compared to older mice [102]. Therefore, administration of osteocalcin in older animals (12 and 15 months old) was sufficient to reverse the age-induced decrease of exercise capacity, conferring to them the same ability of three-month-old animals.

In a brief communication, Mera et al. reported that the weights of the quadriceps, soleus and extensor digitorum longus (EDL) are reduced in 12-month-old female mice lacking the ocn gene, leading to reduced muscle cross-sectional area and body weight. The same phenotype is observed in gprc6a KO, even when the gene is knocked down specifically in myofibers (gprc6a _Mck_-/-), demonstrating that osteocalcin regulates muscle mass independently of its effects on energy expenditure [96]. Indeed, gprc6a _Mck_-/- did not show alteration of glucose tolerance and insulin sensitivity compared to control mice [102]. Accordingly, gprc6a _Mck_-/- mice showed reduced muscle performance during an endurance test [102]. The authors demonstrated that osteocalcin directly promoted protein synthesis in myotubes, explaining why this hormone is responsible for muscle maintenance during aging [96].

**Figure 2:**
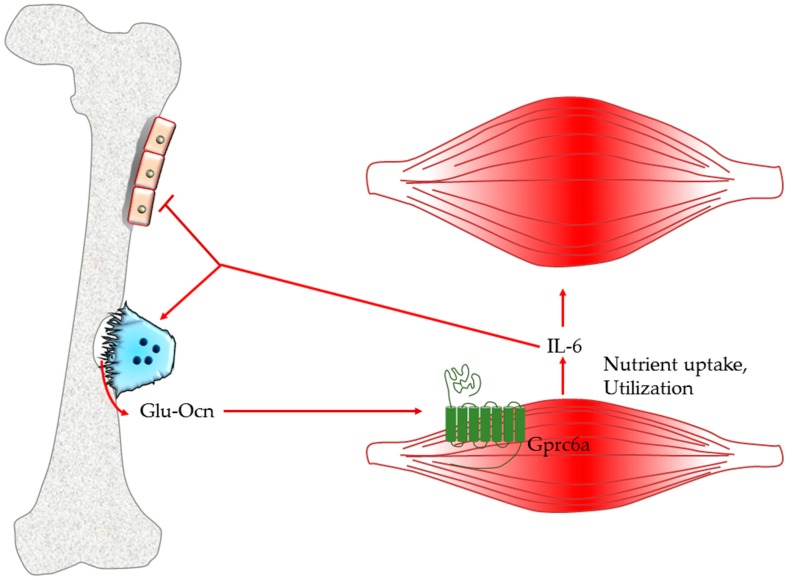
Osteocalcin. The undercarboxylated form of osteocalcin is released during bone resorption. The hormone binds its receptor, gprc6a, expressed on muscle cells stimulating nutrient uptake and the secretion of interleukin 6. In turn, IL-6 reduces osteoblast activity, stimulates bone resorption and induces increase of muscle mass in response to exercise.

Interestingly, treatment with exogenous osteocalcin on cell-specific gprc6a-/- myotubes did not correct the poor ability of the mice to perform exercise, highlighting osteocalcin as the main ligand of this receptor and responsible for the regulation of the muscle response to exercise. These observations explain why exogenous osteocalcin restores the exercise capacity of 15-month-old mice to that of 3-month-old mice [102]. In addition to its regulation of nutrient uptake and catabolism, osteocalcin signaling in myofibers is also responsible for most of the exercise-induced increase of circulating interleukin-6 (IL-6), a myokine that promotes adaptation to exercise in part by increasing the production of bioactive osteocalcin (Figure 2) [102]. Hence, a feed-forward regulation linking together the endocrine functions of bone and muscle appears to be necessary and sufficient to favor adaptation to exercise.

To translate these results into humans, Kim et al. published the results obtained in an exercise program of 8 weeks of 39 obese young men. They demonstrated that, after exercise, body fat decreased, insulin resistance improved and both serum total and undercarboxylated osteocalcin significantly increased [103]. Moreover, in a cross-sectional study Fernández-Real et al. reported that weight loss by caloric restriction and regular exercise increased serum osteocalcin levels, and fat mass was the parameter that best predicted the change in serum OC [104].

Lin et al. performed a study on 24 male college students who were subjected to different exercise programs: a single-bout plyometric exercise group or a 200 m × 10 intermittent running group [105]. An increase of Ocn levels was observed in the plyometric group at 5 min and 1 h after exercise, while no differences were revealed for TRAP (tartrate-resistant acid phosphatase) levels. The authors conclude that, because the increase of osteocalcin was observed after just 5 min of exercise, this could be due to the effects of mechanical loading rather than the activation of bone cells [105]. However, in this study the levels of bone resorption markers were not evaluated, and TRAP did not reveal osteoclast activation.

Levinger et al. demonstrated that in women over the age of 70 years, the percentage of ucOC, but not absolute OC concentration, was positively associated with hip flexor, hip abductor and quadriceps muscle strength. Moreover, the level of total osteocalcin was positively associated with P1NP (type I procollagen N-terminal pro-peptide) and CTX (C-terminal telopeptide of type 1 collagen) [106].

Ahn et al. showed the effects of the exercise (1 h per day, 3 times per week, for 12 weeks) in 29 elderly female subjects classified into normal, osteopenia and osteoporosis groups. The differences between bone mineral content, bone mineral density and osteocalcin concentrations increased significantly in the osteoporosis group after 12 weeks of exercise and were significantly higher than those in the normal and osteopenia groups [107].

The possibility to use osteocalcin as a therapeutic approach is emerging. A recent paper published by Lin X et al. tested the treatment with undercarboxylated osteocalcin in mice receiving a short-term administration of glucocorticoids (GCs) [108]. It was demonstrated that not only the long-term but also the short-term administration of GCs resulted in the development of skeletal muscle insulin resistance, which is the initiating and primary defect of T2DM (type II diabetes mellitus) [108,109,110]. The authors showed that administration of glucocorticoid for three days was sufficient to suppress circulating levels of total and undercarboxylated osteocalcin [108]. Ex vivo ucOC treatment on muscle isolated from GC-treated mice improved muscle insulin sensitivity, acting in a muscle-specific manner and differing between glycolytic (as extensor digitorum longus, EDL) and oxidative (as soleus) muscle [108]. Indeed, it was observed that in EDL muscle, osteocalcin enhanced p-AktSer473 via the increase in total Akt expression; in the soleus, it simulated the phosphorylation of PKC (Protein kinase C), which may lead to enhanced insulin-stimulated activation of Akt and AS160 [111].

## 4. Conclusions

The relationship between bone and muscle is essential for the physiology of the entire body. Indeed, to counteract aging characterized by osteopenia and sarcopenia, maintenance of the correct interaction between these two endocrine organs is necessary. The muscle–bone relationship is so tight that diseases affecting one have detrimental consequences on the other organ.

Understanding the molecular mechanisms underlying this crosstalk could be very important for the identification of therapeutic approaches to face osteopenia and sarcopenia. A very promising molecule is the undercarboxylated osteocalcin that displays antiaging features. Indeed, a surprising result was obtained with the administration of osteocalcin in older animals that allowed them to acquire physical abilities of younger mice. However, many studies were published using animal models, and they need to be confirmed in humans. Some differences between murine and human osteocalcin should be considered. Osteocalcin is γ-carboxylated on glutamic acids (GLU) 13, 17 and 20 of protein in mouse, and on GLU 17, 21 and 24 in humans [83]. Moreover, regarding the circadian rhythm, OC levels in mice peak during the daytime and are at their lowest during nighttime, whereas in humans, the levels fall in the early morning, rise in the afternoon and peak at night [112].

The possibility to use osteocalcin as a therapeutic approach is very promising not only for sarcopenia but also for male fertility and cognitive alterations. Since it was demonstrated that osteocalcin has also positive effects on Leydig cells and the brain, this molecule could be defined as an antiaging tool. Indeed, muscle function, male fertility and cognitive features decline in parallel with osteocalcin levels during aging. If an osteocalcin-dependent approach could be translated into humans, this will open the way for a revolutionary, multitarget approach to counteract the deleterious consequences of aging.
